# Gene flow and genetic structure in Nile perch, *Lates niloticus*, from African freshwater rivers and lakes

**DOI:** 10.1371/journal.pone.0200001

**Published:** 2018-07-11

**Authors:** Rose K. Basiita, Kyall R. Zenger, Matthew T Mwanja, Dean R. Jerry

**Affiliations:** 1 Centre for Sustainable Tropical Fisheries and Aquaculture, College of Science and Engineering, James Cook University, Townsville, Queensland, Australia; 2 National Agricultural Research Organization, National Fisheries Resources Research Institute, Aquaculture Research and Development Center Kajjansi, Kampala, Uganda; 3 WorldFish Zambia Office, Ridgeway Lusaka, Zambia; National Cheng Kung University, TAIWAN

## Abstract

**Background:**

Geological evolution of the African continent has been subject to complex processes including uplift, volcanism, desert formation and tectonic rifting. This complex geology has created substantial biogeographical barriers, and coupled with anthropogenic introductions of freshwater fishes, has influenced the genetic diversity, connectivity and sub-structuring of the teleost fauna. Nile perch, *Lates niloticus*, is an iconic fish in Africa and is of high commercial importance, both in the species’ native range and where it has been translocated. However, the species is in decline and there is a need to understand its population genetic structure to facilitate sustainable management of the fishery and aquaculture development.

**Methodology:**

Nile perch tissue samples were acquired from two West and four East (Lakes; Albert, Kyoga, Victoria and Turkana) African locations. Nineteen polymorphic microsatellite loci were used to study the genetic variation among populations across regions (West and East Africa), as well as between native and introduced environments within East Africa.

**Principal findings and their significance:**

Results revealed strong and significant genetic structuring among populations across the sampled distribution (divergence across regions, *F*_*CT*_ = 0.26, P = 0.000). STRUCTURE analysis at a broad scale revealed K = 2 clusters, the West African individuals were assigned to one cluster, while all individuals from the East African region, regardless of whether native or introduced, were assigned to another cluster. The distinct genetic clusters identified in the current study between the West and East African Nile perch, appear to have been maintained by presence of biogeographic barriers and restricted gene flow between the two regions. Therefore, any translocations of Nile perch should be carefully considered across the regions of West and East Africa. Further analysis at a regional scale revealed further structuring of up to K = 3 genetic clusters in East African Nile perch. Significantly (P < 0.05) lower genetic diversity based on analysis of allelic richness (*A*_*R*_*)* was obtained for the two translocated populations of Lake Kyoga (*A*_*R*_ *= 3*.*61*) and Lake Victoria (*A*_*R*_ = 3.52), compared to Nile perch populations from their putative origins of Lakes Albert (*A*_*R*_ = 4.12) and Turkana (*A*_*R*_ = 4.43). The lower genetic diversity in the translocated populations may be an indication of previous bottlenecks and may also indicate a difficulty for these populations to persist and adapt to climatic changes and anthropogenic pressures that are currently present in the East African region.

## Introduction

Advances in the knowledge-base of African freshwater fisheries have been made over the last five decades [1–3]. Like elsewhere in the world, these advances in knowledge have been driven mainly by threats that are facing the sustainable exploitation of fisheries resources. For African fisheries, these threats include increases in urbanisation linked to human population growth, exotic fish introductions, overfishing, and sedimentation / pollution as a result of changes in land utilisation. The impacts of these threats are particularly noticeable in the freshwater lake basins of East Africa [3–5]. Most freshwater fish research has focused on the evolution of one group of African fishes, the haplochromine cichlids (Cichlidae) in the Great Lakes region. Several mutually contradictory reports have characterised the haplochromine cichlids’ high level of endemism and population demise as a result of human-induced impacts [6–10]. However, many other African species remain data deficient in the face of declining fisheries. This poses a serious threat to the economic development of African communities that largely depend on fish in these lakes and rivers for their livelihoods [11–13].

The genus *Lates* comprises fish from 11 species which occur in both brackish and freshwater, and are widely distributed across Africa, Asia and Australia [14]. *Lates niloticus*, the Nile Perch, is a freshwater species endemic to African rivers and lakes including the Nile, Chad, Senegal, Niger and Congo River basins. This species also naturally occurs in the East African Rift (EAR) Lakes of Tanganyika, Albert and Turkana. However, more recently the range of Nile perch was expanded through anthropogenic introductions about 50–60 years ago by British colonialists, to include Lakes Nabugabo, Kyoga and Victoria [12, 15–17].

Stocking reports for Nile perch are contradictory, but generally indicate that the species was introduced into Lakes Victoria and Kyoga in the 1950s and 1960s (number of stocked individuals ranging from 8–585), where the species has now become established [9, 12, 16–21]. Nile perch introduced into Lake Kyoga putatively originated from Lake Albert, whilst fish introduced into Lake Victoria originated from both Lakes Albert and Turkana in the western and eastern arms of the EAR respectively [16]. Introduced populations have become important to the food security and economic development of communities, with Nile perch introduced into Lake Victoria, for example, being the major commercial freshwater fishery in Africa which supports directly and indirectly up to four million people in East Africa [13]. The fishery from Lake Victoria, contributes up to USD 350 million (from EU exports alone) to the GDP of the three East African countries (Uganda, Kenya and Tanzania) [13]. Nevertheless, there is disagreement among biologists, conservations and economists with regard to the social, environmental and economic impacts of Nile perch introduction to these lakes [16, 17, 21–23].

While anecdotal observations and official records provide a historical understanding of the pattern of introduction of Nile perch into various lakes within the East African region [16], there is still some controversy and limited knowledge of the actual biogeography of the Nile perch in Africa. Information on the genetic structure of Nile perch in Africa at a broad-scale, has been limited to mitochondrial DNA (mtDNA) analyses in which a single West African population (from the Senegal River), and six East African populations were sampled to assess the evolutionary history of the species [24]. In their study, Mwanja et al (2013) identified two historical genetic groupings between East and West Africa, with no shared haplotypes between these two genetic groups [24]. Anectodal information from earlier studies on East African Nile perch populations, using mtDNA and nine microsatellites developed from a sister species, *L*. *calcarifer*, showed at least two genetic subpopulations of Nile perch [20, 25–27]. Fragmented genetic studies limited by the scale of sampling and use of few microsatellite loci, may compromise concrete deductions on the genetic structure and diversity of Nile perch in Africa. Therefore, a holistic Nile perch study to genetically characterise the species across its range in Africa and the clarification of the present genetic stocks using sufficient robust markers was critical.

The current study investigated the population genetic structure of the Nile perch in six populations from Western and Eastern Africa. Unravelling the genetic structure of Nile perch will be useful in understanding how the interactions between biological, physical and geological processes have shaped populations of the species in its natural and introduced range. Information on the broad-scale genetic structure of Nile perch will be critical in achieving successful aquaculture establishments, conservation and management of the wild fisheries.

## Methods

### Study area

Nile perch were sampled over a wide geographical range in Western and Eastern Africa to capture patterns of both biogeographical and human mediated gene flow of the species in both native and introduced environments. Following animal ethics guidelines, the sampling formed part of RKB’s PhD study approved by the animal ethics committe at James Cook University under approval number A1824. All samples for the study were freshly caught fish acquired from commercial fishers as part of their normal fishing activities and outside protected areas and as such we did not require special permits to get these samples. In Western Africa, two locations were sampled, the Senegal River (n = 18) and Lake Kainji, on the Niger River (n = 30). The samples from Senegal River were acquired from fishers and traders at Richard toll (located at the southern bank of Senegal River) in Senegal, while our collaborator from the Federal College of Freshwater Fisheries Technology organised the samples from commercial fishers on Lake Kainji. Similarly, the Eastern African samples were acquired from the species’ native habitats of Lakes Albert (n = 48) and Turkana (n = 18), as well as from introduced populations from Lakes Kyoga (n = 38) and Victoria (n = 38). Important to note is that this study was limited to Western and Eastern African populations, as samples could not be obtained from north of Lake Albert (Albert Nile) to the lower parts of the Nile where the habitat gets brackish towards the Mediterranean Sea.

#### Senegal river basin

The Senegal River, covers 1.6% (483,181 km^2^) of the African continent spreading over four countries (Senegal, Mauritania, Mali and Guinea). The river basin which drains into the Atlantic ocean has been considered as the lifeline of the westernmost part of the Sahel zone of Africa, with agriculture and fishing as the two major economic activities [28, 29]. Nile perch samples for this study were captured below Dagana, at Richard-Toll, where the Senegal enters its delta with the Diama dam. The dam was constructed in 1985 and currently acts as a low water storage dam and salt-water barrier near the mouth of the Senegal River [29].

#### Lake Kainji on river Niger

Lake Kainji located in north-western Nigeria is the largest man-made lake in Western Africa formed in 1968 by damming part of the Niger River. The lake covers an area of up to 1,300 km^2^. Lake Kainji (from which the samples were obtained in the current study) is important and extensively used for fishing and irrigation in the region [30, 31].

#### Lake Albert

Lake Albert is located at the tip of the eastern arm of the EAR. Lake Albert drains directly into the White Nile, to which it is believed to be the main regulating water source for the Nile responsible for making the Nile a permanent river as opposed to a seasonal river [32–36]. The lake is connected to Lake Kyoga to the east by the Victoria Nile and connected to Lake Edward in the south by the Semliki River.

#### Lake Kyoga

Lake Kyoga is centrally located in Uganda. Lake Kyoga is connected to Lakes Victoria and Albert by the Victoria Nile River; however, despite the connection the Lakes are separated by a series of cascading falls, the biggest and most significant being the Murchison falls located just before the Victoria Nile drains into Lake Albert. At the top of the falls, the Victoria Nile narrows to a width of about 7 meters and drops by a height of about 120 metres forming a formidable barrier to upstream movement of aquatic animals. Geologically the Murchison falls were formed as a result of riverine retreat from a buried fault line and superposition from deeply lateralised rift surface sediments [36]. Similarly, to the south of the Victoria Nile is the Owen falls and Rippon falls which separate Lake Kyoga from Lake Victoria. The falls on the northern and southern parts of the Victoria Nile River act as biogeographic barriers across the three water bodies restricting the free movement of fish species, including the Nile perch [20].

#### Lake Victoria

Lake Victoria is the largest tropical freshwater lake in the world and is home for the majority of Africa’s diverse haplochromine species. Currently three commercial species are heavily fished in the lake; *L*. *niloticus* (Nile perch), *Oreochromis niloticus* (Nile tilapia) and *Rastrineobola argentea* (Silver fish). Although introduced into Lake Victoria, Nile perch is the primary fished species for both local consumption and export [16]. Due to heavy fishing pressure Nile perch populations have sharply declined in the lake over the last decade and are becoming threatened [12, 13].

#### Lake Turkana

Turkana is a large holomictic endorheic rift valley lake located in the eastern arm of the EAR; the lake is generally considered to be understudied, but biologically and environmentally stable [37, 38]. Historically, there is evidence that Lake Turkana in the western arm of the EAR was linked until recently (7,000 years ago) to the Nile river system [38, 39]. The lake is central to the livelihoods in the arid northern Kenya and southern Ethiopia providing both fisheries and water resources.

### Sample preservation

Samples from all the six sampling locations were obtained from commercial line fishers, except for the samples from Lake Victoria which were captured by trawling. From each individual fish, a fin clip was cut from the caudal fin and preserved in 20% dimethyl sulfoxide (DMSO) saturated with sodium chloride salt [40, 41], or in 70% ethanol. All fin clips were transferred in fresh DMSO salt solution prior to being shipped to the Molecular Ecology and Evolution Laboratory [42] in Townsville, Australia, where they were stored at -20°C prior to DNA extraction.

### DNA extraction, Polymerase Chain Reaction (PCR) and genotyping

Total genomic DNA was extracted using the Bioline Isolate II Genomic DNA kit (Bioline) following the manufacturer’s protocol. Briefly; the protocol involved a pre-lysis stage using 180 μl Lysis buffer with 25 μl of protease K incubated at 56°C for 1 hr. A second lysis buffer, G-3 (200 μl) was added and further incubated at 70°C for 10 min. In order to alter the buffer conditions, 210 μl of 100% EtOH were then added prior to two (GW1 and GW2) buffer washes. DNA was then eluted using a warm (70°C) elution buffer and later the eluted DNA was stored at -80°C prior to downstream PCR. DNA quality and quantity were assessed using a ND-1000 Spectrophotometer (Nano-Drop^®^ Technologies).

#### Polymerase Chain Reaction

DNA from 192 individuals were genotyped at 19 polymorphic microsatellite loci: 10 markers were species-specific (used in two suites; NP3 and NP1B each of four and six markers respectively) and nine markers (in a single suite, P1 suite) were from a sister *Lates* species; barramundi, *L*. *calcarifer* [43, 44]. For all markers, the forward primers were fluorescently labeled using the 5-dye system (6-FAM, VIC, NED, PET and LIZ GS-500 size standard) and reverse primers were pigtailed [45] to ensure consistent amplification and minimize stuttering. A total volume of 10 μl PCR reactions were run on a Biorad C1000 thermocycler under the following conditions: an initial denaturation at 95°C for 5 min, 6 cycles of 95°C for 30 s (denaturation)/59°C for 90 s (annealing)/72°C for 30 s (extension), 10 cycles at reduced annealing temperatures of 57°C, 55°C and 53°C, prior to a final extension at 60°C for 30 min.

### Genotyping

Visualisation of PCR product was performed on an ABI-3730 instrument (Applied Biosystems) using a 5-standard dye system (6-FAM, VIC, NED, PET and LIZ GS-500 size standard) at the Georgia Genomics Facility, USA. Alleles were scored using Genemarker 2.4 (Softgenetics) and checked for genotyping errors, stuttering and null alleles in Microchecker 2.2.3 [46]. Successful PCR amplification was achieved in all individuals, except for two samples that failed to amplify at several loci. These two individuals were consequently removed in all downstream analyses.

#### Genetic diversity among populations

Deviations from Hardy-Weinberg equilibrium (HWE) and locus linkage equilibrium were evaluated using GenAlex 6.41 [47] and Fisher's Exact test in Genepop on the web v4.2[48] In order to identify genetically diverse populations, diversity indices including expected heterozygosity (*H*_*e*_), observed heterozygosity (*H*_*o*_), mean number of alleles (*N*_*a*_), allelic richness (*AR*), rare and private alleles were calculated in GenAlEx 6.41 [47]. Additionally, allelic richness and rare alleles, while adjusting for small sample size, were calculated in FSTAT and HP Rare Version 1.1, respectively [49, 50]. Furthermore, the Wilcoxon sign- ranked test was performed between population pairs of diversity indices including private alleles for native and translocated populations to test for reductions in genetic diversity that may occur following translocation of populations from their native to new environments. All populations were tested for deviations in heterozygosity using the inbreeding coefficient, *F*_*IS*_, calculated in Genetix v 4.05 [51].

A heterozygosity excess test for recent demographic history for the Nile perch for each of the six populations was executed in Bottleneck 1.2.02 [52]. Although the stepwise mutation model (SMM) is consensually the accepted mutation model for microsatellites assuming that they evolve by addition or subtraction of one or more repeat units, the infinite stepwise model (IAM) was additionally considered in the analyses given that sometimes there could be violations to the SMM. Therefore, the IAM becomes a sensitive model in such scenarios [53]. Moreover in the current study, the two-phase model (TPM) in the proportion of 7:3 (IAM: SMM) was used and differences tested using the Wilcoxon test. The allele frequency mode shift was also explored to determine the populations that have undergone a bottleneck or recent population reduction. Additionally, effective population sizes (*N*_*e*_) for each population, were estimated using the linkage disequilibrium method as implemented in NeEstimator v 2 [54].

#### Genetic differentiation and population structure

Standard genetic tests were performed to examine the amount of genetic differentiation among the six sites sampled. Using an analysis of molecular variance [55], the level of genetic variance partitioned among groups (East and West African populations), among populations, individuals within populations and that within individuals was determined. A subsequent AMOVA test was then performed, excluding samples from the two West African locations to test for the levels of genetic variance in the four East African populations. Global *F*_*ST*_ testing was used to determine whether significant genetic differentiation existed between populations. Following the significant overall *F*_*ST*_ values obtained (at P < 0.001), pairwise genetic differentiation tests were performed on all the six populations after Weir and Cockerham [56]. AMOVA and pairwise *F*_*ST*_ estimates were all calculated in Arlequin 3.5 [57] and Genepop [48], with estimates of population differentiation derived from 10,000 to 16,000 permutations of the data. In order to correct for multiple comparisons and the increased probability of committing a type one error, P-values for pairwise *F*_*ST*_ were corrected using Bonferroni correction at P < 0.001 [58].

To test whether Nile perch populations conform to an isolation by distance (IBD) model of gene flow, genetic distance (pairwise *F*_*ST*_) and geographic distance (km) matrices were constructed and correlations analysed using a Mantel’s test (1000 permutations) in GenAlex 6.41 [47]. Two IBD analyses were performed, one including all the six populations from West and East Africa, and the second analysis, considering only East African Nile perch populations.

The Bayesian assignment test implemented in STRUCTURE 2.3.3 [59] was used to determine the number of inferred genetic clusters and to test the proportion of genetic admixture among all the six populations. The implementation of individual-based clustering in this program creates clusters of individuals within which the Hardy-Weinberg equilibrium is maximised and linkage disequilibrium minimised, thus assigning individuals into gene pools, or breeding groups [60]. To evaluate the number of genetic clusters, analyses involved testing up to 10 possible clusters (from K = 1 to K = 10), followed by 10 iterations using the admixture model with the assumption of correlated allele frequencies and no location prior information provided. Similar tests of the admixture model were performed, but this time assuming that allele frequencies are independent; except for this modification all other input parameters in STRUCTURE analyses were left the same (i.e. a burn-in period of 100,000 iterations and 100,000 MCMC). The result files from the STRUCTURE were zipped and uploaded into a web based program, STRUCTURE HARVESTER, which uses the methods described by Evanno, Regnaut [61]. STRUCTURE HARVESTER processses and outputs the most distinct number of clusters K, and the corresponding most probable run among the runs for eack K value. Further visual representation of gene flow and relative migration among populations was performed using divMigrate analyses in the *R* diveRsity package [62]. The visual network derived depicts arrows that indicate the direction and magnitude of gene flow between populations.

## Results

### Genetic diversity indices and differentiation

Microchecker did not detect any evidence of scoring errors due to stuttering and large allelic dropouts at any of the loci examined. Linkage disequilibrium was observed in only 0.05% (8 out of 172) of loci combinations across all populations, following Bonferroni correction set at P < 0.001 (S1 Table). However, deviations from HWE were observed at three loci among the translocated Nile perch populations of Lakes Kyoga and Victoria from East Africa. These loci were; *Ln11* (in samples from Lake Kyoga), *Ln23* (samples from Lake Victoria) and *Ln31* (samples from lakes Kyoga and Victoria). These deviations from HWE (at P < 0.001 after Bonferroni correction) may suggest a detection of non-random mating within the two introduced populations.

**Fig 1:**
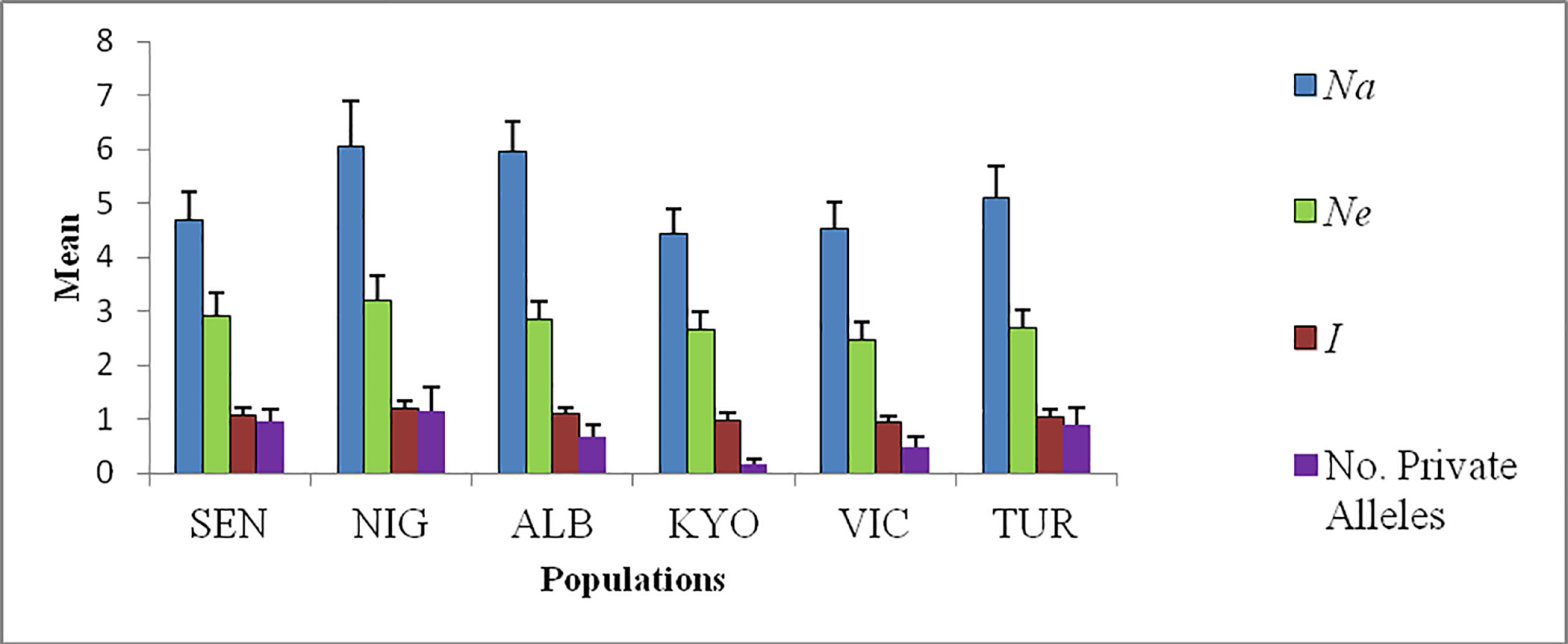
Mean allelic patterns in Nile perch plotted against each population. The Y–axis, shows the mean number of alleles (*Na*), *Ne*, mean number of effective alleles and Mean number of private alleles, while the X- axis shows the populations; SEN—Senegal, NIG—Lake Kainji on the Niger River, ALB- Lake Albert, KYO- Lake Kyoga, VIC- Lake Victoria and TUR- Lake Turkana. Error bars represent standard error (SE).

Most loci were polymorphic across populations, except for *Ln09* and *Ln23*, which were monomorphic in the Lake Turkana population, and *Lca21* which was also monomorphic in the Lake Kainji (Niger) population. The mean number of alleles (*N*_*a*_) per locus for all six populations was 5.13 ± 0.02 (mean ± SE) and number of alleles in the polymorphic loci ranged from 2–14 alleles (Table 1). Among East African Nile perch populations, private alleles were highest in native Nile perch populations, but low in the translocated populations of Lakes Kyoga and Victoria (Fig 1).

**Table 1:** Microsatellite DNA statistics for *Lates niloticus* from six populations in West and East Africa.

Location	*N*	No. of alleles	*N*_*a*_ ± SE (range)	*A*_*R*_ ± SE (range)	*H*_*o*_ ± SE (range)	*H*_*e*_ ± SE (range)	*F*_*IS*_
**Senegal River**	18	89	4.68 ± 0.53 (2–9)	4.31 ± 0.47 (2–9)	0.56 ± 0.66 (0.14–0.94)	0.54 ±0.05 (0.18–0.87)	0.009*
**Lake Kainji -Niger River**	30	115	6.05 ± 0.84 (1–14)	4.88 ± 0.58 (1–11)	0.52 ± 0.07 (0.00–0.92)	0.55 ±0.06 (0.00–0.87)	0.078*
**Lake Albert**	48	113	5.95 ± 0.57 (3–12)	4.19 ± 0.40 (2–9)	0.45 ± 0.05 (0.09–0.78)	0.54 ±0.05 (0.09–0.86)	0.148*
**Lake Kyoga**	38	84	4.42 ± 0.49 (2–8)	3.61 ± 0.35 (1–7)	0.51 ± 0.06 (0.03–0.87)	0.51 ±0.06 (0.03–0.82)	0.019*
**Lake Victoria**	38	86	4.53 ± 0.49 (2–10)	3.52 ± 0.35 (1–8)	0.50 ± 0.06 (0.03–0.87)	0.48 ±0.06 (0.03–0.84)	0.002*
**Lake Turkana**	18	97	5.11 ± 0.57 (1–9)	4.43 ± 0.458 (1–8)	0.54 ± 0.08 (0.00–1.00)	0.51 ±0.06 (0.00–0.83)	-0.029*

Values for microsatellite statistics are means over all loci (range) for each location: *N*, sample size, *N*_*a*_, mean number of alleles, *A*_*R*_, allelic richness, *H*_*o*_, mean observed heterozygosity, *H*_*e*_, mean expected heterozygosity and *F*_*IS*_, inbreeding coefficient.

* Significant *F*_*IS*_ values at P< 0.05.

The allelic richness (*A*_*R*_) across all 19 loci in the six populations ranged from 2.37 at *Lca21* to 9.54 at locus *Ln17* (Table 1). The *A*_*R*_ of native populations across the two regions of West and East Africa were comparable with a narrow range (*A*_*R*_ = 4.19 ± 0.40 to A_R_ = 4.88 ± 0.58 for Lake Albert in East Africa and Lake Kainji in West Africa, respectively). Analysis of *A*_*R*_ as a measure of genetic diversity found a significantly (P < 0.05) lower *A*_*R*_ among individuals from introduced (Lakes Kyoga and Victoria) populations than in individuals from the putative origins for fish introduced (Lakes Albert and Turkana) in East Africa (Table 1). Similarly, the private alleles were lowest in the translocated Kyoga and Lake Victoria populations and highest in native Lake Albert and Turkana populations of Nile perch (Fig 1). A similar trend was observed for the rare alleles where there was a statistical difference between the introduced and native Nile perch populations within East African Nile perch.

The overall mean expected and observed heterozygosities (Mean ± SE) were 0.51 ± 0.03 and 0.52 ± 0.02, respectively across all populations. There were no large variations in heterozygosity observed amongst populations, although Lake Albert showed the lowest mean *H*_*o*_ (0.45 ± 0.05) and Lake Victoria the lowest *H*_*e*_ (0.48 ± 0.06). All diversity indices, including the mean expected and observed heterozygosity, for each population across all loci are listed in Table 1.

Lake Turkana exhibited the only negative mean *F*_*IS*_ value of– 0.029, while Lake Albert had the highest positive *F*_*IS*_ of 0.148. The high *F*_*IS*_ within Lake Albert could be a result of sub-structuring of the Nile perch within the lake, and this is consistent with the admixture observed in this Albert population as per the STRUCTURE bar plot. Results of the bottleneck tests did not detect significant deviations in allele frequencies, and in all instances an L-shaped curve was obtained (results not shown). However, with the Wilcoxon test, significant heterozygosity deficiencies were identified within the native populations of Nile perch from Lake Albert and Turkana (P = 0.004) under the IMM model and for Lake Kyoga a heterozygote excess was identified under the IAM model (P = 0.03). The effective population size (*Ne*) at the lowest frequency of 0.05 ranged from (*Ne* = 20.3; 95% CI 15–27.5) in Lake Kainji to (*Ne* = 253; 95% CI 38.4-∞) in the Senegal River. Effective population size, *Ne*, in East African populations ranged from *Ne* = 39.6; 95% CI 28.8–58.8) in Lake Albert to *Ne* = 79.1; 95% CI 45.5–213) in Lake Victoria. Except for the Senegal population, in all other populations the *Ne* was finite and restricted.

#### Genetic differentiation and structure

Nile perch were found to show strong genetic structure, especially between West and East African populations (Global *F*_*ST*_ of 0.26, P < 001) (Table 2). Genetic variation among the six populations was partitioned into various sources, with total genetic variance predominantly due to that within individuals (70.29%) and lowest was between groups (3.27%) (Table 2). Pairwise *F*_*ST*_ between populations across the West and East African region were very high and significant (*F*_*ST*_ range of 0.34 to 0.42, P<0.001), although the *F*_*ST*_ were relatively shallow among populations within groups (*F*_*ST*_ range of 0.07 to 0.20, significant P < 0.001) (Table 3 and S2 Table).

**Table 2:** Groupings for the Analysis of Molecular Variance (AMOVA) for *Lates niloticus* at 19 microsatelite loci.

Source of variation	Sum of squares	Variance components	Percentage variation
Among Populations	547.52	1.81	26.44
Among individuals within populations	885.83	0.22	3.27
Within individuals	840	4.80	70.29
Total	2273.34	6.83	
FST: 0.26			

**Table 3:** Estimates of pairwise *F*_*ST*_ between six Nile perch populations.

	Senegal River	Kainji (Niger River)	Lake Albert	Lake Kyoga	Lake Victoria	Lake Turkana
**Senegal River**	-	*	*	*	*	*
**Kainji (Niger River)**	0.14	-	*	*	*	*
**Lake Albert**	0.38	0.34	-	*	*	*
**Lake Kyoga**	0.39	0.35	0.13	-	*	*
**Lake Victoria**	0.42	0.40	0.08	0.17	-	*
**Lake Turkana**	0.42	0.40	0.13	0.21	0.18	-

Below diagonal is *F*_*ST*_ and significance (sequential Bonferroni corrected P-value ≤0.001) denoted by an * symbol above diagonal

The high and significant *F*_*ST*_ indicate major differences in allele frequencies between the East and West African populations. The highest pairwise genetic differentiation was between the Senegal River and the two most geographically distant East African populations of Lake Turkana and Lake Victoria (*F*_*ST*_ = 0.42, P < 0.001), which were the most geographically distantly sampled populations. The lowest pairwise *F*_*ST*_ was between Lake Victoria and Albert (*F*_*ST*_ = 0.08), although it was still significant (P = 0.000 after sequential Bonferroni correction).

**Fig 2:**
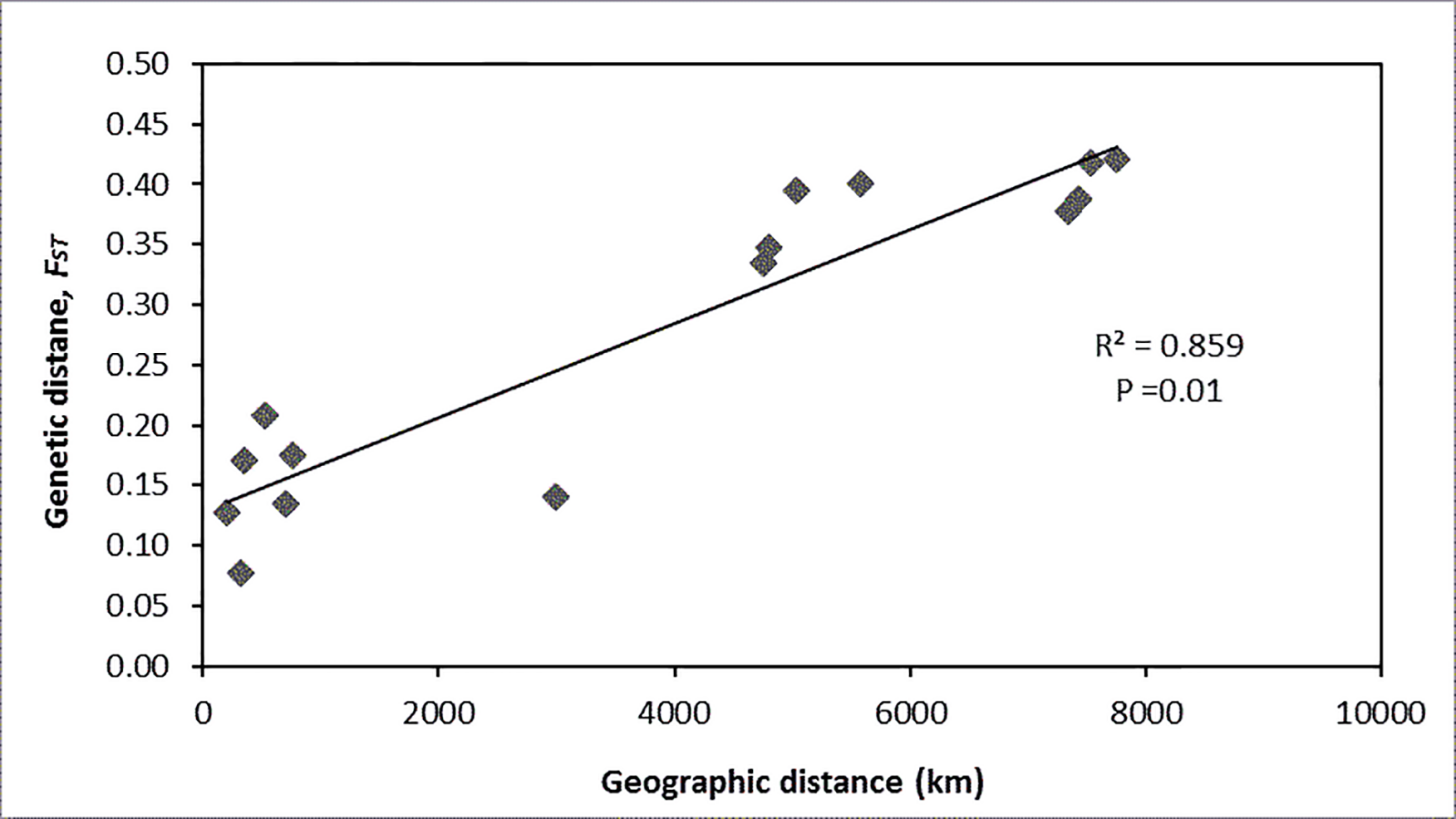
Mantels test comparing geographic (km) and genetic (*F*_*ST*_) distance of Nile perch, *Lates niloticus*, populations across six locations in West and East Africa (n = 190).

Furthermore, the Mantel’s test revealed a positive and significant correlation (R^2^ = 0.86, P = 0.01) (Fig 2). This significance seemed to have been driven by the strong differentiation of West African samples from the East African samples in the analysis, since at a within East African regional scale there was a very low and non-significant correlation (R^2^ = 0.165, P = 0.2).

**Fig 3:**
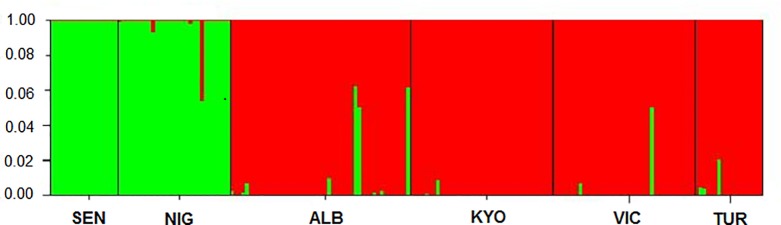
STRUCTURE bar plot showing the genetic assignment (K = 2) of West (Green) and East (red) African Nile perch, *Lates niloticus*, populations. The X-axis represents the sampling locations SEN- Senegal, NIG- Lake Kainji on the Niger River, ALB- Lake Albert, KYO- Lake Kyoga, VIC- Lake Victoria and TUR- Lake Turkana.

Bayesian STRUCTURE analysis confirmed strong partitioning of genetic varian ce among Nile perch populations with two distinct genetic clusters (K = 2) identified (Fig 3). The individuals from West Africa (Senegal and Lake Kainji on Niger River) were assigned to one genetic group (green cluster) and the East African populations (Lakes Albert, Kyoga, Victoria and Turkana) mainly to another (red) (Fig 3), although in a few cases individuals with some sharing of genetic ancestry were evident. All individuals from the Senegal River were only assigned to the green genetic cluster (West) and no admixture was observed in this population, unlike the population from Lake Kainji on the Niger River that consisted of a few individuals with an admixed green and red genome ancestry.

### Gene flow and relative migration among western and eastern Nile perch populations

**Fig 4:**
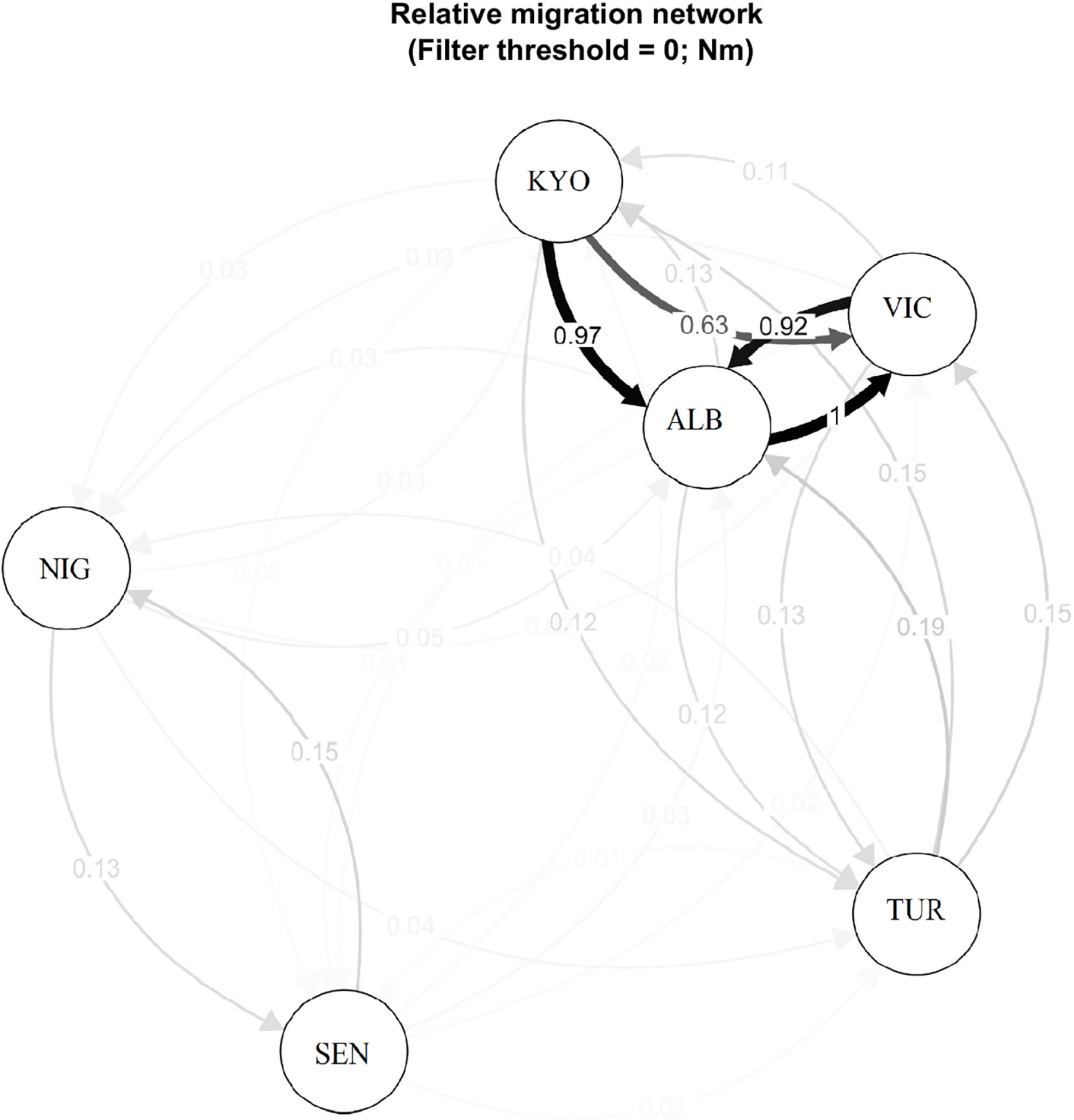
Relative migration network diagram for Nile perch, *Lates niloticus* from six sample locations in West and East Africa. Circles represent sample locations;—SEN- Senegal, NIG -Nigeria, ALB—Albert, KYO—Kyoga, VIC -Victoria and TUR -Turkana. The arrows contain edge values showing the direction and magnitude of migration levels, with the darker arrows showing stronger gene flow. The figure reveals stronger gene flow between populations ALB- Albert (putative population) and VIC- Lake Victoria (translocated population originally from mainly Lake Albert) albeit reduced gene flow between Lake Albert and Lake Kyoga (another translocated population also from Lake Albert).

Results showed relational connectivity, reflecting the directional and relative strengths of migration between the six sampled populations of Nile perch. The Nile perch from the Senegal River and Lake Kainji clustered into one genetic group and all the other four populations into another major genetic group. The two major genetic groups exhibited in the divMigrate relative gene flow network diagram (Fig 4), did mirror the STRUCTURE assignment genetic clusters (Fig 3). Here, restricted gene flow was detected between the West and East African populations. Additionally, the gene flow between the two West African populations was low compared to the gene flow among the East African populations.

Similarly, there is a genetic sub-group exhibited among three (Lakes Albert, Kyoga and Victoria) of the four East African populations shown by the high gene flow between these populations. However, restricted gene flow was shown to and from the Lake Turkana population to the rest of the East African populations; Turkana is appropriately excluded by geographical distance and historical connectivity. In a way, the relative migration network did show genetic separation of the populations relative to distance, but more so was a surrogate of historical Nile perch translocations that have characterised the species from its native (Lakes Albert and Turkana) to the new introduced (Lake Kyoga and Victoria) environments. Important to note, is that some locations had lower sample numbers (N<30) which may underrepresent the potential diversity in these particular locations. Possible sampling sites affected in the present study may include the Niger River, Senegal River and Lake Turkana.

### Population sub-structuring within East and West African Nile perch populations

**Fig 5:**
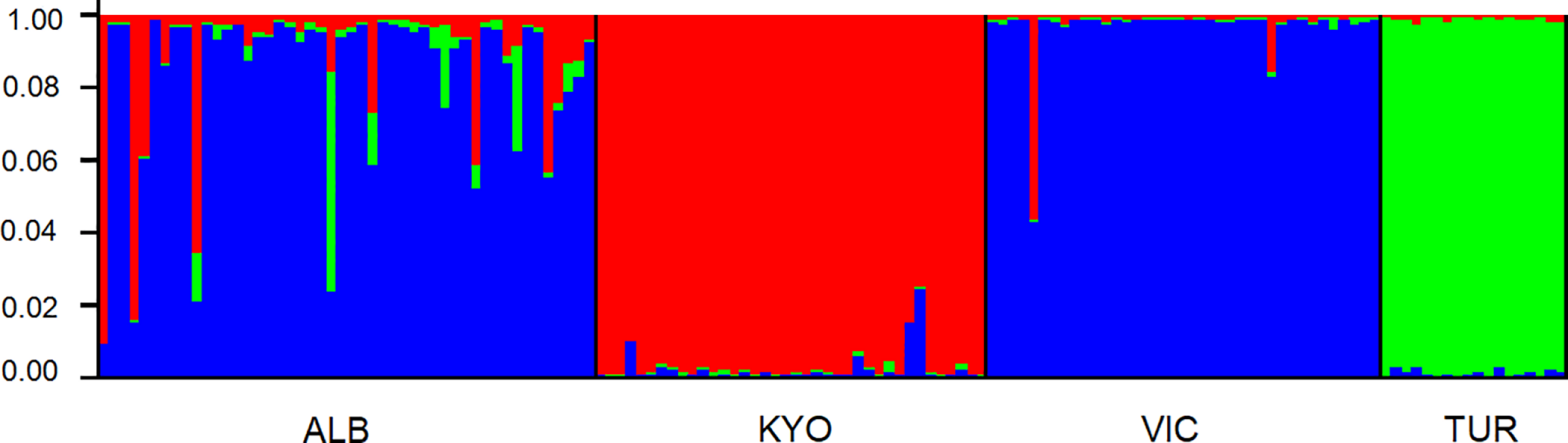
STRUCTURE [59] bar plot for Nile perch stock of East Africa populations; ALB—Lake Albert, KYO—Kyoga, VIC -Victoria and TUR—Turkana. The bar plot shows the proportion of genome ancestry assigned to three genetic clusters represented by blue, red and green colours. Lake Victoria and Albert individuals were assigned to one major cluster (blue) and Kyoga (red), while Turkana to another separate cluster (green).

Regional STRUCTURE analysis also revealed strong genetic structuring among the EAR lake populations, with three distinct genetic clusters evident (Fig 5); the Lake Turkana cluster (green), Lake Kyoga (red), and Lake Victoria (blue). Lake Albert individuals were mainly assigned to the same genetic grouping as Lake Victoria fish, although Nile perch in Lake Albert exhibited evidence of increased genetic admixture with fish from Lakes Kyoga and Turkana.

Similarly, further structuring was observed in the samples from West Africa, where individuals from Senegal River were assigned to a different genetic group from the Nigerian samples of Lake Kainji on the Niger River. This result is consistent with the significant, albeit shallow *F*_*ST*_ value obtained between the Senegal and Nigerian individuals sampled in the current study (Table 3). It is important to not that sample size for both of these populations was low (> 50) and such results should be interpreted with precaution.

## Discussion

The Nile perch in Africa was partitioned into two discrete genetic groupings that may require different management strategies; one from West Africa and the other from East Africa. Further structuring within East Africa was revealed, with three discrete populations at a regional scale. The strong genetic structure is a function of restricted levels of gene flow between populations across biogeographical barriers, particularly for the Nile perch in East Africa. Barriers including river flow and reversal, dams and water falls have been identified as being effective in impeding gene flow across connected large water bodies. For instance, to the right of Lake Albert are the Murchison Falls along the Victoria Nile River, a biogeographical barrier separating the Albertine rift system (includes Lakes Edward, George and Albert) from Lake Victoria. This barrier has been documented as an effective obstacle in preventing, for instance, Nile perch stocks in Lake Albert from migrating into Lakes Kyoga and Victoria [63]. Important to note though, is that for the Nile perch in East Africa, the structuring may also partially be a product of historical anthropogenic introductions and translocations among the different water bodies [9, 16]. Furthermore, Mwanja et al. [20] reports genetic sub-structuring within Lake Victoria suggesting at least two seperate introductions from different sources. The categorical evidence of decreased genetic diversity exemplified within the translocated populations of Lake Victoria and Kyoga suggest introgression and loss of genetic diversity resulting from anthropogenic activities. The two translocated Nile perch populations of Lake Kyoga and Lake Victoria are genetically distinct and ought to be treated separately for sustainable management of the now dwindling fishery in the two new habitats. However, for an aquaculture breeding program within the East African region, all three distinct populations should be included at the start of the breeding program to maximise the advantages of diverse genetic variability given that the *F*_*ST*_ values within regions were shallow, albeit significant (P = 0.000).

### Broad scale genetic differentiation and structure

The Nile perch in Africa’s freshwater rivers and lakes was highly differentiated with significant (P < 0.000) global *F*_*ST*_ of 0.26 and strong genetic structuring which is comparable to other freshwater species with similar dispersal and habitat usage [64]. Because freshwater habitats are typically discrete (unless connected by river systems), populations occupying such habitats tend to display strong genetic structuring due to isolation and restricted gene flow. Nile perch in Africa was confirmed to be partitioned into two discrete genetic groupings; one from West Africa and the other one in East Africa. Separate genetic groupings among populations can only occur following restricted gene flow over time [65]. Therefore, the existence of the two major Nile perch groups confirmed in the current study using high resolution nuclear markers clearly indicates the separation of these genetic lineages over a long period of time.

West African and East African genetic groupings from the current study are further supported based on a single study carried out in Nile perch from the broader Africa region using a single mitochondrial marker (d-loop region) [24]. That study identified two genetic groupings of the Nile perch in Africa, with no shared haplotypes between them, which diverged during the late Pleistocene [24]. The detection of strong genetic differentiation (at both the mtDNA and nuclear DNA markers) between the West and East African populations of Nile perch indicates that the Nile perch, like other species (e.g. African buffalo, *Syncerus caffer*, and Nile tilapia *Oreochromis niloticus)* in Africa, exhibits a genetic break between the populations of Western and Eastern Africa [66, 67].

The significant isolation by distance revealed by the Mantel’s test in the current investigation further suggests limited dispersal of the species between the two regions of West and East Africa. The result is consistent with findings in other aquatic species, where patterns of isolation by distance have been found to arise from limited dispersal that decreases with increased distance [68–74]. Strong genetic differentiation is found to be common over a larger geographical area as opposed to minimal or no structuring at all between populations in close geographic proximity, although recent findings reveal a discrepancy between dispersal and genetic structuring [71, 75]. Thus, whereas the IBD seemed to be playing a role in the separation of the two major genetic groupings of West and East Africa, further analysis at a regional scale revealed that the Nile perch in East Africa does not conform to IBD.

### Genetic differentiation and structure in East Africa

The genetic structure in East Africa is complex and the current study unraveled up to three differentiated genetic clusters which appeared to be grouped according to lakes. Except for Lakes Victoria and Albert that were relatively undifferentiated from each other, there was comparatively deeper differentiation observed amongst all the other lakes in the East African region (all with significant *F*_*ST*_, P<0.000). Strong genetic structuring among the East African populations was evident where Lakes Albert and Victoria clustered together (Group 1); and Lake Kyoga with Lake Turkana each were assigned to their own genetic cluster (Group 2 and 3 respectively). Results of the current study showing three genetic groupings differs from an earlier study that identified only two genetic groupings within the East African lakes (25). How the three genetic groupings have evolved in the face of biogeographic events and geological structures is still open to interpretation, but these three genetically distinct Nile perch populations identified for the first time in the current study may be treated as different management units (based on their high level of differentiation and reduced connectivity) for the sustainable exploitation of the species.

It is hypothesized that human mediated introductions of Nile perch into new habitats and the complex geological history in the East African region are some of the key factors driving the genetic structure and evolution of this and other species in the region. Previous studies in other freshwater species in the region have demonstrated that the complex geological history in East Africa is a major driver of diversification at both species and population levels [6, 76, 77]. However, the structuring within the East African Nile perch populations seems to be in agreement to some degree with reports of the anthropogenic introductions of the Nile perch into Lake Victoria and Kyoga, with parent introduced Nile perch stocks derived from Lake Albert and a few individuals from Lake Turkana [16].

### Genetic structure following fish introductions in the East African freshwater lakes

In the current study, strong genetic differentiation was detected among native and translocated Nile perch. As mentioned earlier, the major translocations in the region involved the stocking of Nile perch from Lakes Albert and Turkana into Lakes Victoria and Kyoga [11, 16, 78]. Although a portion of Nile perch individuals introduced into Lake Victoria were putatively acquired from Lake Turkana, results from the current investigation showed that the Nile perch in Lake Victoria exhibited a very limited genetic signature from Lake Turkana, but had a strong signal from Lake Albert. The very weak signal from Lake Turkana seems to be consistent with the historical stocking records, where only eight individuals were introduced in Lake Victoria at Kisumu Port [18]

In addition to the Nile perch individuals from Lake Victoria exhibiting a strong genetic signal from the Lake Albert population, there was also evidence of strong directional gene flow obtained for the species between these two lakes (Fig 5). This could be due to the fact that; 1) multiple introductions of Nile perch from Lake Albert were made into Lake Victoria [16] and 2) the niches in Lake Victoria are highly variable, and some of these habitats could still mimic to a certain degree those available in the source habitat of Lake Albert. Lake Victoria has varied habitats that harbour Nile perch, while displacing other species [habitats include shallow and deep water characterised by a gradient of hypoxic, chronic hypoxic and anoxic conditions [79–81]]. The genetic convergence of Nile perch from Lake Victoria and Albert is slightly contradictory to an earlier report that was based on morphology, indicating that the form of Nile perch in Lake Victoria differed from the form in the putative parental origins of Lake Albert and Turkana [82]. The results in the current study clearly show that the Nile perch in Lake Victoria is still genetically similar to the Nile perch in Lake Albert, with shallow but significant *F*_*ST*_ (P = 0.003), although these populations have become divergent from Lake Turkana individuals (*F*_*ST*_ = 0.18, P = 0.003; Table 3, Fig 4) which lies in the eastern side of the EAR [82].

Nile perch from Lake Kyoga was differentiated from that of Lake Albert, a finding which is quite interesting since the foundation stocks into Lake Kyoga were derived from Lake Albert (Fig 5). This is possibly due to low founder numbers that may have survived following stocking and possibly most genotypes to the red genetic ancestry (Fig 5) may have survived and consequently propagated. Another possibility could be the uncertainties usually associated with facts on fish introductions, in which case the historical record of introduction from Lake Albert may be questionable, and it is likely that the stock could have been sourced from elsewhere, probably in the upper Nile. Unfortunately in the current investigation, there were no samples acquired from the Nile river system to ascertain this possibility, and as such we propose that future genetic work should involve samples from the Nile River [16].

### Genetic diversity

Globally, introductions of non-native fish species into freshwater ecosystems are a major concern. This is especially so in the African Great Lakes region, where fish translocations have supposedly resulted in disruptions to aquatic biodiversity and overall community structure. Moreover, the translocated populations may go through bottlenecks that are revealed through reduced genetic diversity. Low genetic diversity has been found to impact negatively on the viability of populations posing concerns for conservation as populations may fail to adapt and persist in the event of harsh ecological and environmental pressures [83, 84].

The current study showed evidence of reduced genetic diversity in the translocated Nile perch populations of Lakes Victoria and Kyoga, compared to the native populations from which the founder stocks were derived. The higher genetic diversity in the native Nile perch populations of Lakes Albert and Turkana, indicates that these populations have a higher potential to adapt and persist over time in their native habitats, than would be the introduced populations [84–86]. The findings in the current investigation are contrary to earlier findings in the same species where the native populations showed lower genetic diversity (allelic diversity) than the translocated populations [20]. However, data in the present study should be viewed as being more robust, as the genetic differences were statistically significant and population sample sizes were considered with regards to diversity indices. Furthermore the patterns observed in the current study for the allelic diversity are consistent with the general hypothesis of loss of genetic diversity due to founder effects within translocated populations (e.g. in the speckled dace cyprinid fish, *Rhinichthys osculus* which was introduced into Van Duzen River in California [87].

The situation of reduced genetic diversity in the introduced Nile perch is exacerbated by pressures that the Nile perch could be experiencing in the new environments (of Lakes Kyoga and Victoria) in comparison to their putative original populations (from Lakes Albert and Turkana). These include; heavy fishing pressure coupled with climate and ecological pressures which may in part be responsible for the current declines of Nile perch in Lakes Kyoga and Victoria. Results from the present study are consistent with reports that have shown declining numbers of Nile perch in Lakes Kyoga and Victoria despite the increase in fishing effort of the Nile perch amidst climate and ecological pressures [13, 17, 18, 20, 45, 88, 89]. Nile perch declines were first detected in Lake Kyoga as early as the 1980’s, and predictions of such declines speculated then for Lake Victoria have now been realised [88].

Although reduced genetic diversity was detected among the translocated populations at the allelic level, the genetic bottleneck was not severe enough to be detected by the bottleneck analysis [90, 52]. This non-detection was most probably due to the multiple introductions that generally could have counter balanced the founder effects that were not severe enough to be detected by the heterozygosity method employed under bottleneck analysis.

### Management implications

Knowledge of genetic structure and relationships revealed in the present study among Nile perch populations will be useful for the effective management and conservation of the species in both its native and introduced environments. Separate management strategies of Nile perch populations from West and East Africa (for both aquaculture development and fisheries restocking) should be considered, following the genetic divergence and differentiation that has occurred over evolutionary time between these two groups. Following restricted gene flow, the two genetic groupings have evolved in isolation, and consequently may have accrued adaptive differences, and any translocations of Nile perch should be carefully considered across the regions of West and East Africa.

Programs aimed at restocking these lakes and rivers should seek founder stocks independently within each of the two regions of West and East Africa. As discussed previously, there was complex structuring within East African Nile perch where three distinct genetic stocks were identified, and these seemed to be linked to lakes. Thus, within East Africa, the Nile perch from Lake Albert and Lake Victoria should be treated as a single group, Lake Kyoga as another and Turkana separately. Movement of the Nile perch across lake basins should be discouraged to prevent possible genetic introgression.

Furthermore, from an aquaculture perspective, the recognition of the three genetic groupings identified within East Africa, provides a large genetic base upon which genetic diversity can be maximized through potentially mixing the three distinct genetic populations at the start of a Nile perch breeding program for the region. However, it is important to note that even though the populations are distinct, *F*_*ST*_ is relatively shallow and could potentially be mixed together without raising concerns of out breeding that may include a reduction in fitness [64]. The three genetic groups may hold a broad range of standing genetic variation, which aquaculture efforts (through breeding programmes) can capitalise on to develop an artificial base population. A similar approach was utilized in the development of the GIFT strain of Tilapia, where individuals from various sources of up to eight strains were mixed together to provide a foundation for a genetic improvement program [91, 92].

## Conclusion

Nile perch in Africa is partitioned into two discrete genetic groupings; one comprising populations from West Africa and the other populations in East Africa. Further structuring within East Africa was revealed, with three discrete populations requiring different management strategies at a regional scale. Although, strong genetic structure is a function of restricted levels of gene flow between populations across biogeographical barriers, in the current study, results indicated that it may also partially be a product of historical anthropogenic introductions. The categorical evidence of decreased genetic diversity exemplified the persistence of founder effects within the translocated populations of Lakes Victoria and Kyoga. The two translocated Nile perch populations of Lakes Kyoga and Victoria were distinct, and ought to be treated separately for sustainable management of the now dwindling fishery in the two new habitats. The use of more robust markers in the current study clearly resolved the genetic structure of Nile perch in East Africa, and the three populations identified will be useful in forming an artificial and diverse base population for selective breeding of the Nile perch. Therefore, findings and information provided by the current study will be critical and useful in achieving successful and comprehensive aquaculture establishments, as well as conservation and management of the wild fisheries for the freshwater Nile perch.

## Supporting information

S1 Table Linkage disequilibrium between loci combinations across all populations. Significant deviations following a sequential Bonferroni (at P<0.001) correction are indicated by sig and non-significant by ns.
(DOCX)

S2 Table Pairwise *F*_*ST*_ estimates for *Lates niloticus*, Nile perch, for all loci as estimated in Genepop on the web 1.2 [48]. SEN- Senegal River, NIG- Lake Kainji, ALB- Lake Albert, KYO- Lake Kyoga, VIC–Lake Victoria and TUR- Lake Turkana. All pairwise comparisons were significant at P<0.05
(DOCX)

S3 Table P-value for each population pair across all loci (Fisher's method) implemented in Genepop v 4.2. (DOCX)

S4 Table Genetic diversity tests for Nile perch, *Lates niloticus*, performed in FSTAT vs 2.9.3.2. a) **Allelic Richness per locus and population**
b) **Number of alleles sampled**
c) **Nei's estimation of heterozygosity.**
(DOCX)

S1 Graph Delta K for Nile Perch samples from East Africa. (PDF)

S2 Graph Structure bar plot for West African samples, Nigeria and Senegal. (TIF)
