# Supplementary material for: Gene flow and genetic structure in Nile perch, Lates niloticus, from African freshwater rivers and lakes
Source: PLoS One. 2018 Jul 11;13(7):e0200001. doi: 10.1371/journal.pone.0200001 (PMC6040733; doi:10.1371/journal.pone.0200001)
Supplement: S1 Table — Significant deviations following a sequential Bonferroni (at P<0.001) correction are indicated by sig and non-significant by ns. (DOCX) [file pone.0200001.s001.docx]

| **Locus Pair** | **P- value** | **Significance after BF correction (P<0.001)** |
| --- | --- | --- |
| *Lca08 - Lca21* | 0.473845 | ns |
| *Lca08 - Lca69* | 0.134888 | ns |
| *Lca08 - Lca70* | 0.856118 | ns |
| *Lca08 - Lca98* | 0.631919 | ns |
| *Lca08 - Ln09* | 0.751508 | ns |
| *Lca08 - Ln10* | 0.754913 | ns |
| *Lca08 - Ln11* | 0.871627 | ns |
| *Lca08 - Ln31* | 0.889322 | ns |
| *Lca20 - Lca08* | 0.53766 | ns |
| *Lca20 - Lca21* | 0.679519 | ns |
| *Lca20 - Lca70* | 0.488531 | ns |
| *Lca20 - Lca98* | 0.867584 | ns |
| *Lca20 - Ln09* | 0.593766 | ns |
| *Lca20 - Ln10* | 0.484361 | ns |
| *Lca20 - Ln11* | 0.139973 | ns |
| *Lca20 - Ln31* | 0.281037 | ns |
| *Lca21 - Lca70* | 0.083587 | ns |
| *Lca21 - Lca98* | 0.09418 | ns |
| *Lca21 - Ln09* | 0.909244 | ns |
| *Lca21 - Ln10* | 0.823577 | ns |
| *Lca21 - Ln11* | 0.725201 | ns |
| *Lca21 - Ln31* | 0.596987 | ns |
| *Lca58 - Lca08* | 0.995697 | ns |
| *Lca58 - Lca20* | 0.5959 | ns |
| *Lca58 - Lca21* | 0.737602 | ns |
| *Lca58 - Lca69* | 0.28715 | ns |
| *Lca58 - Lca70* | 0.874892 | ns |
| *Lca58 - Lca98* | 0.862074 | ns |
| *Lca58 - Ln09* | 0.211763 | ns |
| *Lca58 - Ln10* | 0.620908 | ns |
| *Lca58 - Ln11* | 0.999849 | ns |
| *Lca58 - Ln31* | 0.432461 | ns |
| *Lca64 - Lca08* | 0.045761 | ns |
| *Lca64 - Lca20* | 0.919774 | ns |
| *Lca64 - Lca21* | 0.093184 | ns |
| *Lca64 - Lca58* | 0.033216 | ns |
| *Lca64 - Lca69* | 0.925175 | ns |
| *Lca64 - Lca70* | 0.444409 | ns |
| *Lca64 - Lca98* | 0.086117 | ns |
| *Lca64 - Ln09* | 0.912414 | ns |
| *Lca64 - Ln10* | 0.994245 | ns |
| *Lca64 - Ln11* | 0.933869 | ns |
| *Lca64 - Ln31* | 0.784254 | ns |
| *Lca69 - Lca21* | 0.680395 | ns |
| *Lca69 - Lca70* | 0.650975 | ns |
| *Lca69 - Lca98* | 0.202709 | ns |
| *Lca69 - Ln09* | 0.627302 | ns |
| *Lca69 - Ln10* | 0.377689 | ns |
| *Lca69 - Ln11* | 0.704024 | ns |
| *Lca69 - Ln31* | 0.095364 | ns |
| *Lca70 - Lca98* | 0.209319 | ns |
| *Lca70 - Ln09* | 0.590476 | ns |
| *Lca70 - Ln10* | 0.253418 | ns |
| *Lca70 - Ln11* | 0.623025 | ns |
| *Lca70 - Ln31* | 0.943863 | ns |
| *Lca74 - Lca08* | 0.936267 | ns |
| *Lca74 - Lca20* | 0.192856 | ns |
| *Lca74 - Lca21* | 0.261683 | ns |
| *Lca74 - Lca58* | 0.538154 | ns |
| *Lca74 - Lca64* | 0.554683 | ns |
| *Lca74 - Lca69* | 0.71156 | ns |
| *Lca74 - Lca70* | 0.804785 | ns |
| *Lca74 - Lca98* | 0.491614 | ns |
| *Lca74 - Ln09* | 0.291263 | ns |
| *Lca74 - Ln10* | 0.119031 | ns |
| *Lca74 - Ln31* | 0.185223 | ns |
| *Lca98 - Ln09* | 0.966977 | ns |
| *Lca98 - Ln10* | 0.290625 | ns |
| *Lca98 - Ln11* | 0.727798 | ns |
| *Lca98 - Ln31* | 0.499494 | ns |
| *Ln02 - Lca08* | 0.56349 | ns |
| *Ln02 - Lca20* | 0.295426 | ns |
| *Ln02 - Lca21* | 0.781242 | ns |
| *Ln02 - Lca58* | 0.387967 | ns |
| *Ln02 - Lca64* | 0.155556 | ns |
| *Ln02 - Lca69* | 0.390843 | ns |
| *Ln02 - Lca70* | 0.357571 | ns |
| *Ln02 - Lca74* | 0.013067 | ns |
| *Ln02 - Lca98* | 0.548307 | ns |
| *Ln02 - Ln09* | 0.710578 | ns |
| *Ln02 - Ln10* | 0.808529 | ns |
| *Ln02 - Ln17* | 0.136822 | ns |
| *Ln02 - Ln23* | 0.239636 | ns |
| *Ln09 - Ln10* | 0.374678 | ns |
| *Ln09 - Ln10* | 0.374678 | ns |
| *Ln11 - Ln09* | 0.774707 | ns |
| *Ln11 - Ln10* | 0.094837 | ns |
| *Ln15 - Lca08* | 0.468595 | ns |
| *Ln15 - Lca20* | 0.509858 | ns |
| *Ln15 - Lca21* | 0.218403 | ns |
| *Ln15 - Lca58* | 0.615616 | ns |
| *Ln15 - Lca64* | 0.271526 | ns |
| *Ln15 - Lca69* | 0.323635 | ns |
| *Ln15 - Lca70* | 0.661926 | ns |
| *Ln15 - Lca74* | 0.85427 | ns |
| *Ln15 - Lca98* | 0.273414 | ns |
| *Ln15 - Ln02* | 0.266995 | ns |
| *Ln15 - Ln09* | 0.996638 | ns |
| *Ln15 - Ln10* | 0.993954 | ns |
| *Ln15 - Ln11* | 0.011413 | ns |
| *Ln15 - Ln23* | 0.053381 | ns |
| *Ln15 - Ln29* | 0.91718 | ns |
| *Ln15 - Ln31* | 0.341141 | ns |
| *Ln17 - Lca08* | 0.61627 | ns |
| *Ln17 - Lca20* | 0.743607 | ns |
| *Ln17 - Lca21* | 0.872606 | ns |
| *Ln17 - Lca64* | 0.645709 | ns |
| *Ln17 - Lca69* | 0.576378 | ns |
| *Ln17 - Lca70* | 0.247501 | ns |
| *Ln17 - Lca74* | 0.417542 | ns |
| *Ln17 - Lca98* | 0.865505 | ns |
| *Ln17 - Ln09* | 0.594995 | ns |
| *Ln17 - Ln10* | 0.578207 | ns |
| *Ln17 - Ln11* | 0.042318 | ns |
| *Ln17 - Ln23* | 0.636463 | ns |
| *Ln19 - Lca08* | 0.997054 | ns |
| *Ln19 - Lca20* | 0.300788 | ns |
| *Ln19 - Lca21* | 0.211957 | ns |
| *Ln19 - Lca58* | 0.722913 | ns |
| *Ln19 - Lca64* | 0.962531 | ns |
| *Ln19 - Lca69* | 0.624461 | ns |
| *Ln19 - Lca70* | 0.865987 | ns |
| *Ln19 - Lca74* | 0.006648 | ns |
| *Ln19 - Lca98* | 0.787921 | ns |
| *Ln19 - Ln02* | 0.472136 | ns |
| *Ln19 - Ln09* | 0.785886 | ns |
| *Ln19 - Ln10* | 0.262912 | ns |
| *Ln19 - Ln11* | 0.300643 | ns |
| *Ln19 - Ln15* | 0.019652 | ns |
| *Ln19 - Ln17* | 0.726416 | ns |
| *Ln19 - Ln23* | 0.583389 | ns |
| *Ln19 - Ln29* | 0.656511 | ns |
| *Ln19 - Ln31* | 0.621049 | ns |
| *Ln23 - Lca08* | 0.141831 | ns |
| *Ln23 - Lca20* | 0.282408 | ns |
| *Ln23 - Lca21* | 0.269076 | ns |
| *Ln23 - Lca58* | 0.298156 | ns |
| *Ln23 - Lca64* | 0.189877 | ns |
| *Ln23 - Lca69* | 0.650719 | ns |
| *Ln23 - Lca70* | 0.527844 | ns |
| *Ln23 - Lca74* | 0.778772 | ns |
| *Ln23 - Lca98* | 0.41623 | ns |
| *Ln23 - Ln09* | 0.457027 | ns |
| *Ln23 - Ln10* | 0.635062 | ns |
| *Ln23 - Ln11* | 0.20239 | ns |
| *Ln23 - Ln31* | 0.517196 | ns |
| *Ln29 - Lca08* | 0.389832 | ns |
| *Ln29 - Lca20* | 0.035329 | ns |
| *Ln29 - Lca21* | 0.390994 | ns |
| *Ln29 - Lca58* | 0.947353 | ns |
| *Ln29 - Lca64* | 0.797242 | ns |
| *Ln29 - Lca69* | 0.126584 | ns |
| *Ln29 - Lca70* | 0.71274 | ns |
| *Ln29 - Lca74* | 0.707676 | ns |
| *Ln29 - Lca98* | 0.551725 | ns |
| *Ln29 - Ln02* | 0.02523 | ns |
| *Ln29 - Ln09* | 0.23468 | ns |
| *Ln29 - Ln10* | 0.905091 | ns |
| *Ln29 - Ln11* | 0.193666 | ns |
| *Ln29 - Ln17* | 0.996391 | ns |
| *Ln29 - Ln31* | 0.158705 | ns |
| *Ln31 - Ln09* | 0.240827 | ns |
| *Ln31 - Ln10* | 0.457154 | ns |
| *Ln31 - Ln11* | 0.427965 | ns |
| *Lca20 - Lca69* | Highly sig. | sig |
| *Lca74 - Ln11* | Highly sig | sig |
| *Ln02 - Ln11* | Highly sig | sig |
| *Ln02 - Ln31* | Highly sig | sig |
| *Ln15 - Ln17* | Highly sig | sig |
| *Ln17 - Lca58* | Highly sig | sig |
| *Ln17 - Ln31* | Highly sig | sig |
| *Ln29 - Ln23* | Highly sig | sig |
