# Supplementary material for: Gene flow and genetic structure in Nile perch, Lates niloticus, from African freshwater rivers and lakes
Source: PLoS One. 2018 Jul 11;13(7):e0200001. doi: 10.1371/journal.pone.0200001 (PMC6040733; doi:10.1371/journal.pone.0200001)
Supplement: S2 Table — SEN- Senegal River, NIG- Lake Kainji, ALB- Lake Albert, KYO- Lake Kyoga, VIC–Lake Victoria and TUR- Lake Turkana. All pairwise comparisons were significant at P<0.05 (DOCX) [file pone.0200001.s002.docx]

| **Population** | **SEN** | **NIG** | **ALB** | **KYO** | **VIC** | **TUR** |
| --- | --- | --- | --- | --- | --- | --- |
| **SEN** |  |  |  |  |  |  |
| **NIG** | 0.1248 |  |  |  |  |  |
| **ALB** | 0.3580 | 0.3092 |  |  |  |  |
| **KYO** | 0.3864 | 0.3285 | 0.1089 |  |  |  |
| **VIC** | 0.3991 | 0.3666 | 0.0678 | 0.1462 |  |  |
| **TUR** | 0.4002 | 0.3738 | 0.1308 | 0.1916 | 0.1735 |  |
