# Supplementary material for: Gene flow and genetic structure in Nile perch, Lates niloticus, from African freshwater rivers and lakes
Source: PLoS One. 2018 Jul 11;13(7):e0200001. doi: 10.1371/journal.pone.0200001 (PMC6040733; doi:10.1371/journal.pone.0200001)
Supplement: S3 Table — (DOCX) [file pone.0200001.s003.docx]

| Population pair | | Chi2 | df | P-Value |  |
| --- | --- | --- | --- | --- | --- |
| SEN | NIG | Infinity | 38 | Highly | sign. |
| SEN | ALB | Infinity | 38 | Highly | sign. |
| NIG | ALB | Infinity | 38 | Highly | sign. |
| SEN | KYO | Infinity | 38 | Highly | sign. |
| NIG | KYO | Infinity | 38 | Highly | sign. |
| ALB | KYO | Infinity | 38 | Highly | sign. |
| SEN | VIC | Infinity | 38 | Highly | sign. |
| NIG | VIC | Infinity | 38 | Highly | sign. |
| ALB | VIC | Infinity | 38 | Highly | sign. |
| KYO | VIC | Infinity | 38 | Highly | sign. |
| SEN | TUR | Infinity | 38 | Highly | sign. |
| NIG | TUR | Infinity | 38 | Highly | sign. |
| ALB | TUR | Infinity | 38 | Highly | sign. |
| KYO | TUR | Infinity | 38 | Highly | sign. |
| VIC | TUR | Infinity | 38 | Highly | sign. |
