# Supplementary material for: Gene flow and genetic structure in Nile perch, Lates niloticus, from African freshwater rivers and lakes
Source: PLoS One. 2018 Jul 11;13(7):e0200001. doi: 10.1371/journal.pone.0200001 (PMC6040733; doi:10.1371/journal.pone.0200001)
Supplement: S4 Table — a) Allelic Richness per locus and population b) Number of alleles sampled c) Nei's estimation of heterozygosity. (DOCX) [file pone.0200001.s004.docx]

|  | **SEN** | **NIG** | **ALB** | **KYO** | **VIC** | **TUR** | **Overall** |
| --- | --- | --- | --- | --- | --- | --- | --- |
| *Ln*19 | 2.000 | 2.875 | 2.992 | 2.997 | 2.889 | 3.940 | 6.331 |
| *Ln*15 | 7.012 | 8.984 | 6.401 | 5.136 | 4.952 | 7.442 | 8.199 |
| *Ln*29 | 3.641 | 4.907 | 4.384 | 3.362 | 2.659 | 2.611 | 5.153 |
| *Ln*02 | 7.542 | 7.288 | 5.336 | 4.942 | 4.583 | 4.461 | 8.318 |
| *Ln*17 | 6.479 | 7.624 | 9.148 | 7.344 | 7.791 | 8.307 | 9.539 |
| *Ln*23 | 2.610 | 2.961 | 2.637 | 1.882 | 2.408 | 1.000 | 3.641 |
| *Lca*74 | 5.506 | 5.326 | 7.001 | 5.677 | 6.366 | 6.194 | 8.313 |
| *Lca*64 | 2.733 | 2.352 | 2.062 | 1.289 | 2.018 | 3.467 | 2.435 |
| *Lca*58 | 8.826 | 10.652 | 4.520 | 3.974 | 4.268 | 6.882 | 7.468 |
| *Lca*20 | 2.000 | 4.153 | 3.488 | 3.000 | 3.288 | 2.983 | 4.018 |
| *Lca*08 | 2.669 | 1.393 | 2.899 | 2.945 | 2.000 | 3.750 | 3.492 |
| *Lca*69 | 2.000 | 2.866 | 4.255 | 3.000 | 3.301 | 3.000 | 4.655 |
| *Lca*21 | 3.284 | 1.000 | 1.920 | 2.762 | 1.297 | 3.369 | 2.371 |
| *Lca*70 | 3.000 | 3.655 | 2.846 | 2.945 | 2.000 | 3.600 | 4.650 |
| *Lca*98 | 7.311 | 8.309 | 5.069 | 6.090 | 4.103 | 6.155 | 8.278 |
| *Ln*31 | 2.636 | 5.243 | 3.756 | 2.813 | 3.599 | 7.423 | 5.984 |
| *Ln*11 | 3.610 | 6.287 | 3.979 | 2.591 | 3.086 | 4.136 | 5.806 |
| *Ln*09 | 4.283 | 3.764 | 2.415 | 1.297 | 2.192 | 1.000 | 4.794 |
| *Ln*10 | 4.706 | 3.029 | 4.549 | 4.551 | 4.130 | 4.464 | 4.738 |
| **Mean** | **4.308** | **4.877** | **4.192** | **3.610** | **3.523** | **4.431** | **5.694** |
| **SE** | **0.470** | **0.584** | **0.399** | **0.354** | **0.352** | **0.458** | **0.467** |

**SEN NIG ALB KYO VIC TUR Total**

*Ln*19 2 3 3 3 3 5 11

*Ln*15 8 12 10 6 7 9 22

*Ln*29 4 6 5 4 3 3 8

*Ln*02 8 9 6 5 5 5 14

*Ln*17 7 9 12 9 10 9 17

*Ln*23 3 3 4 2 4 1 5

*Lca*74 6 7 10 8 7 7 16

*Lca*64 3 3 4 2 3 4 6

*Lca*58 9 11 9 5 7 8 24

*Lca*20 2 5 5 3 4 3 5

*Lca*08 3 2 4 3 2 5 8

*Lca*69 2 3 5 3 4 3 5

*Lca*21 4 1 4 3 2 4 7

*Lca*70 3 4 4 3 2 4 8

*Lca*98 8 14 6 7 6 8 24

*Ln*31 3 7 6 5 5 8 16

*Ln*11 4 8 6 4 4 5 12

*Ln*09 5 4 5 2 3 1 9

*Ln*10 5 4 5 7 5 5 8

| **Total** | **89** | **115** | **113** | **84** | **86** | **97** | **225** |
| --- | --- | --- | --- | --- | --- | --- | --- |

**Locus Ho Hs Ht Dst Dst' Ht' Gst Gst' Gis**

*Ln*19 0.398 0.471 0.791 0.321 0.385 0.856 0.406 0.450 0.154

*Ln*15 0.704 0.751 0.846 0.095 0.114 0.865 0.112 0.131 0.062

*Ln*29 0.362 0.548 0.711 0.163 0.195 0.743 0.229 0.263 0.340

*Ln*02 0.775 0.787 0.868 0.081 0.097 0.884 0.093 0.109 0.016

*Ln*17 0.861 0.850 0.891 0.041 0.050 0.900 0.046 0.055 -0.014

*Ln*23 0.231 0.262 0.580 0.318 0.381 0.643 0.548 0.592 0.117

*Lca*74 0.806 0.761 0.870 0.109 0.130 0.892 0.125 0.146 -0.058

*Lca*64 0.145 0.231 0.248 0.017 0.021 0.252 0.069 0.082 0.374

*Lca*58 0.692 0.649 0.801 0.152 0.182 0.831 0.190 0.220 -0.068

*Lca*20 0.662 0.596 0.710 0.113 0.136 0.732 0.160 0.186 -0.110

*Lca*08 0.352 0.339 0.676 0.337 0.405 0.743 0.499 0.544 -0.039

*Lca*69 0.608 0.614 0.748 0.133 0.160 0.774 0.178 0.206 0.011

*Lca*21 0.151 0.182 0.194 0.011 0.014 0.196 0.059 0.070 0.172

*Lca*70 0.452 0.496 0.737 0.241 0.289 0.785 0.327 0.369 0.089

*Lca*98 0.745 0.735 0.868 0.132 0.159 0.894 0.153 0.178 -0.013

*Ln*31 0.470 0.475 0.724 0.248 0.298 0.774 0.343 0.385 0.012

*Ln*11 0.460 0.489 0.664 0.175 0.210 0.699 0.264 0.301 0.060

*Ln*09 0.267 0.268 0.696 0.428 0.514 0.782 0.615 0.657 0.003

*Ln*10 0.619 0.653 0.738 0.085 0.102 0.754 0.115 0.135 0.052

**Overall 0.514 0.535 0.703 0.168 0.202 0.737 0.240 0.274 0.039**
