# Supplementary figures and images for: Gene flow and genetic structure in Nile perch, Lates niloticus, from African freshwater rivers and lakes
Source: PLoS One. 2018 Jul 11;13(7):e0200001. doi: 10.1371/journal.pone.0200001 (PMC6040733; doi:10.1371/journal.pone.0200001)

$$\text{DeltaK} = \text{mean}(|L''(K)|) / \text{sd}(L(K))$$

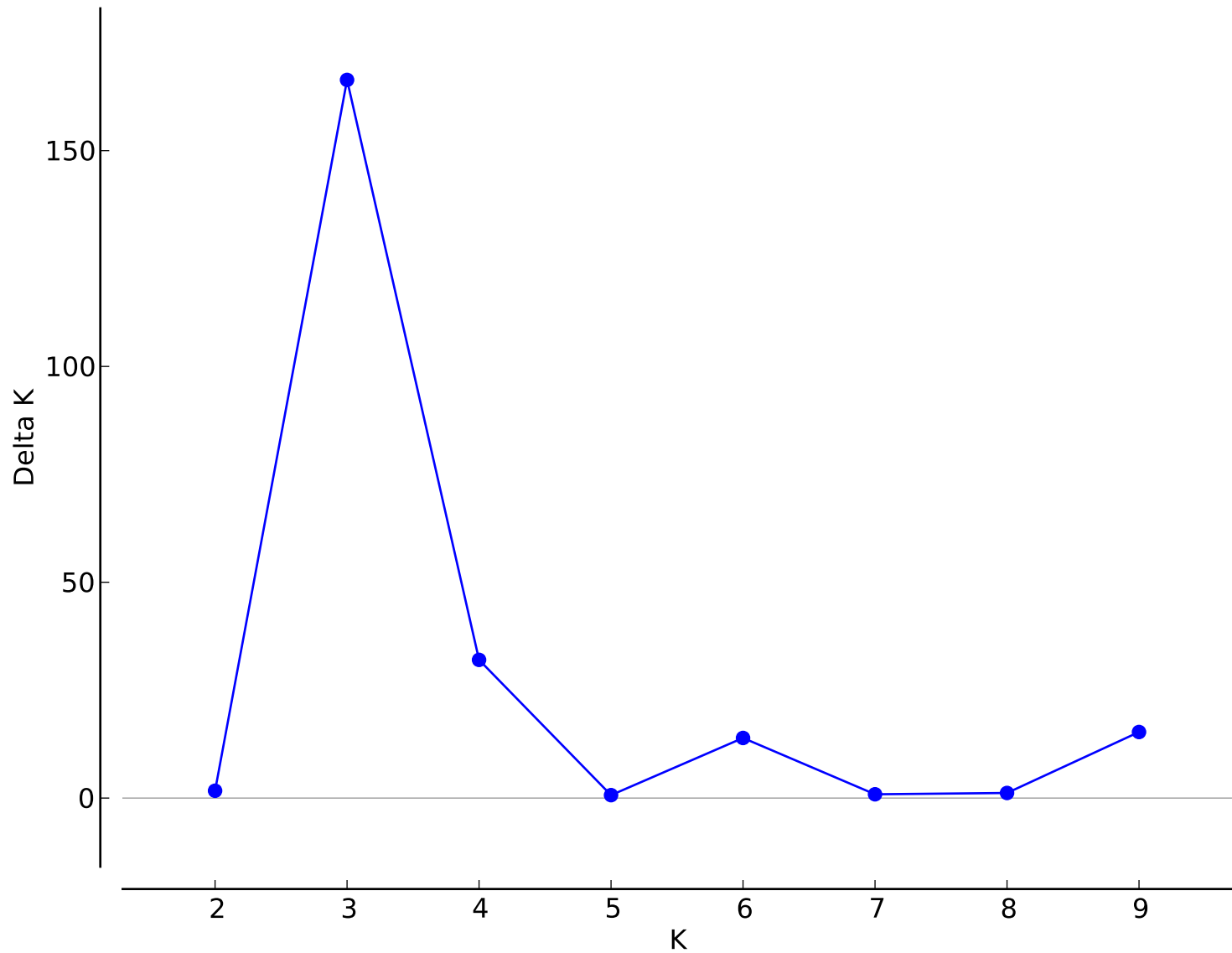

Supplement: S1 Graph — (PDF) [file pone.0200001.s005.pdf]

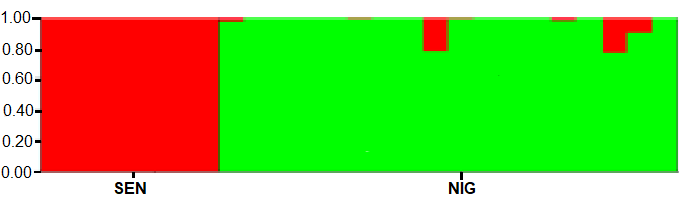

Supplement: S2 Graph — (TIF) [file pone.0200001.s006.tif]
